# Extubation Failure in Critically Ill COVID-19 Patients: Risk Factors and Impact on In-Hospital Mortality

**DOI:** 10.1177/08850666211020281

**Published:** 2021-06-02

**Authors:** Filip Ionescu, Markie S. Zimmer, Ioana Petrescu, Edward Castillo, Paul Bozyk, Amr Abbas, Lauren Abplanalp, Sanjay Dogra, Girish B. Nair

**Affiliations:** 1 Department of Internal Medicine, 21818 Beaumont Health System, OUWB School of Medicine, Royal Oak, MI, USA; 2 Department of Radiation Oncology, 21818 Beaumont Health System, OUWB School of Medicine, Royal Oak, MI, USA; 3 Department of Computational and Applied Mathematics, Rice University, TX, USA; 4 Division of Pulmonary and Critical Care Medicine, 21818 Beaumont Health System, OUWB School of Medicine, Royal Oak, MI, USA; 5 Department of Cardiovascular Medicine, 21818 Beaumont Health System, Royal Oak, OUWB School of Medicine, MI, USA

**Keywords:** critical illness, reintubation, COVID-19, novel coronavirus

## Abstract

**Purpose:**

We sought to identify clinical factors that predict extubation failure (reintubation) and its prognostic implications in critically ill COVID-19 patients.

**Materials and Methods:**

Retrospective, multi-center cohort study of hospitalized COVID-19 patients. Multivariate competing risk models were employed to explore the rate of reintubation and its determining factors.

**Results:**

Two hundred eighty-one extubated patients were included (mean age, 61.0 years [±13.9]; 54.8% male). Reintubation occurred in 93 (33.1%). In multivariate analysis accounting for death, reintubation risk increased with age (hazard ratio [HR] 1.04 per 1-year increase, 95% confidence interval [CI] 1.02 -1.06), vasopressors (HR 1.84, 95% CI 1.04-3.60), renal replacement (HR 2.01, 95% CI 1.22-3.29), maximum PEEP (HR 1.07 per 1-unit increase, 95% CI 1.02 -1.12), paralytics (HR 1.48, 95% CI 1.08-2.25) and requiring more than nasal cannula immediately post-extubation (HR 2.19, 95% CI 1.37-3.50). Reintubation was associated with higher mortality (36.6% vs 2.1%; *P* < 0.0001) and risk of inpatient death after adjusting for multiple factors (HR 23.2, 95% CI 6.45-83.33). Prone ventilation, corticosteroids, anticoagulation, remdesivir and tocilizumab did not impact the risk of reintubation or death.

**Conclusions:**

Up to 1 in 3 critically ill COVID-19 patients required reintubation. Older age, paralytics, high PEEP, need for greater respiratory support following extubation and non-pulmonary organ failure predicted reintubation. Extubation failure strongly predicted adverse outcomes.

## Introduction

In its most severe form, coronavirus disease 2019 (COVID-19) caused by the novel pathogen severe acute respiratory syndrome coronavirus 2 (SARS-CoV-2) can lead to an acute respiratory distress syndrome requiring invasive mechanical ventilation. Once intubated, up to one half of patients exhibit a protracted clinical course marked by prolonged need for mechanical ventilation and less than half of intubated patients are safely liberated from the ventilator during their hospital stay.^1-4^ Furthermore, early reports indicate the success rate of extubation success is low, with as many as 1 in 6 patients requiring reintubation within 7 days.^1,2^

There is currently limited data to characterize the risk of extubation failure (reintubation) in COVID-19 patients. Also, the impact on the risk of extubation failure and on in-hospital mortality of the clinical experience gained since wide adoption of corticosteroids, therapeutic anticoagulation, and antivirals in critically ill COVID-19 patients remains unknown.

In this study, we hypothesize that the risk of extubation failure in COVID-19 is increased by disease severity, either pulmonary (high positive end-expiratory pressure requirement or need for salvage therapies) or extrapulmonary (need for vasopressor support or renal replacement therapy). Accordingly, we performed an observational cohort study in the largest hospital network in Southeast Michigan, USA, examining the extubation failure rate in critically ill COVID-19 patients.

## Methods

### Study Design

We conducted a retrospective analysis of consecutive COVID-19 patients hospitalized at 8 hospitals, forming the largest academic healthcare system in Southeast Michigan, USA. Patients aged 18 years or older, who tested positive for SARS-CoV-2 on nucleic amplification testing of nasopharyngeal secretions between March 12, 2020, and October 19, 2020, on days 1 to 3 of a hospital stay were retrospectively identified from electronic medical records. Only patients who required endotracheal intubation and for whom an extubation attempt was made were included in the analysis. Patient demographics (age, sex, ethnicity), information about therapeutic modalities (anticoagulation, corticosteroids, intubation and mechanical ventilation, dialysis, vasopressor use), comorbid conditions and laboratory data were obtained through automatic data extraction from electronic medical records. The study was approved by the Institutional Review Board (IRB # 2020-125).

**Figure 1.**
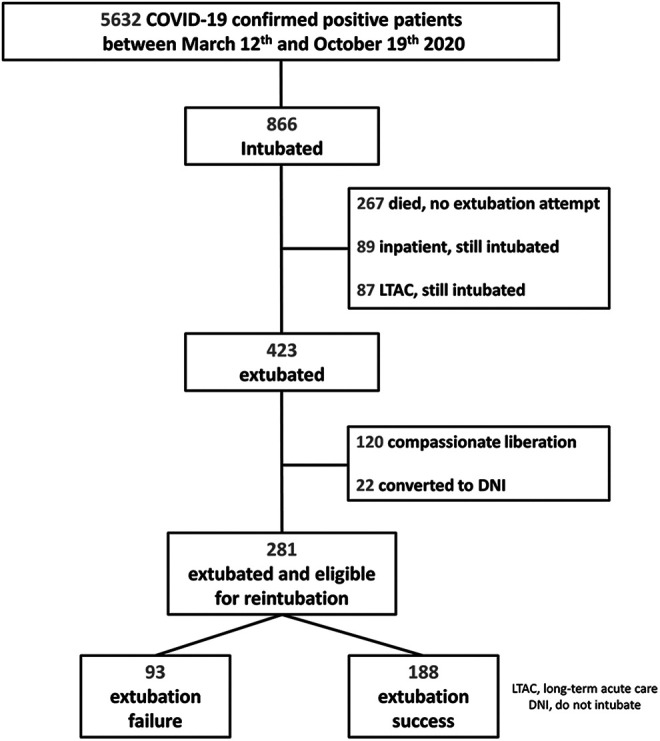
Study population.

Extubation failure was defined as reintubation at any point during the same hospital stay. Those who elected to change code status to no reintubation after extubation were excluded from the analysis (Figure 1) to avoid selection bias. The decision to extubate was based on multidisciplinary ICU team discussion that took into account multiple factors such as success of a spontaneous breathing trial, a calculated rapid shallow breathing index of less than 110, and the need for non-pulmonary organ support such as vasopressors or renal replacement therapy. All intubations were performed by a dedicated COVID-19 team composed of anesthesiologists.

### Outcomes and Statistical Analysis

The main outcome was the reintubation rate which was explored with cumulative incidence graphs and estimates obtained using reintubation as event of interest and death as the competing risk. Gray’s test was used for comparing cumulative incidence curves by variables of interest. Time zero was the time of extubation. Patients who did not experience extubation failure (i.e. were not reintubated) by the end of the study period and did not die were censored at the time of last clinical follow-up. A proportional sub-distribution hazards regression model with reintubation as the event of interest and death as the competing event was performed to assess the impact of candidate variables on the reintubation rate. Candidate variables were preliminarily tested for significance in a univariate Cox proportional hazards model using backward and forward regression.

The secondary outcome was the time to death of any cause compared between successfully extubated patients and those who experienced failure. For this analysis, cumulative incidence graphs and estimates were obtained using death as event of interest and discharge alive as competing risk.^5^ Patients who did not die by the end of the study period and were not discharged alive were censored at the time of last clinical follow-up.

Statistical analysis was performed using JMP version 14 (SAS Institute, Cary, North Carolina) and R (software version 3.5.0). Categorical variables are described as frequency (percentage). Normal or approximately normal variables are reported using the mean (standard deviation), whereas skewed variables are reported with the median (interquartile range [IQR]). Categorical variables were compared using the Chi-square test or Fisher exact test. Normal variables were compared using a 2-sided Student t-test and ordinal variables used the Kruskal-Wallis test. All *P*-values were 2-sided and a *P* < 0.05 was considered to indicate statistical significance.

## Results

### Study Population and Baseline Characteristics

Over 7 months, there were 5632 consecutive patients admitted to our hospital system who tested positive for COVID-19. Intubation and mechanical ventilation were required in 866 (15.4%); 426/866 (49.2%) died inpatient. The endotracheal tube was removed in 423/866, with 303/866 representing extubation attempts (35.0%) and 120/866 (13.9%) representing compassionate liberations from the ventilator. Those who elected to not be reintubated if their clinical status deteriorated again after extubation (22/303) were excluded from the analysis. A total of 281 extubated COVID-19 patients formed the final study population (Figure 1). Extubation failure (reintubation) occurred in 93 (33.1%) patients.

Baseline demographics are presented in Table 1. The average age was 61 ± 13.9 years, with patients failing extubation constituting an older population (66.2 *vs* 58.5 years old; *P* < 0.0001). The 2 groups had a similar sex distribution, but Caucasians accounted for a higher proportion of the reintubation group.

**Table 1.** Baseline Characteristics in Extubated COVID-19 Population.^a^

	All patients (n = 281)	Reintubated (n = 93)	Not reintubated (n = 188)	Significance
Age in years	61.0 (13.9)	66.2 (11.7)	58.5 (14.3)	<0.0001
Male	154 (54.8%)	54 (58.0%)	100 (53.2%)	0.440
*Race*				
African-American	145 (51.6%)	43 (46.2%)	102 (54.3%)	
Caucasian	114 (40.6%)	45 (48.4%)	69 (36.7%)	0.097
Asian	5 (1.8%)	0 (0.0%)	5 (2.7%)	
Other	17 (6.0%)	5 (5.4%)	12 (6.4%)	
BMI categories (kg/m^2^)				0.054
<18.5	2 (0.7%)	2 (2.1%)	0 (0.0%)	
18.5-30	109 (38.8%)	33 (35.5%)	76 (40.4%)	
30-40	114 (40.6%)	44 (47.3%)	70 (37.2%)	
≥40	56 (19.9%)	14 (15.1%)	42 (22.3%)	

^a^Age is presented as mean (standard deviation). Other numbers represent n (%). BMI, body mass index.

Common comorbid conditions recorded on the date of extubation did not significantly differ between groups (Table 2). The Sequential Organ Failure Assessment (SOFA) score was calculated on intubation day and yielded a similar risk of overall mortality (9 [IQR 8-12] for reintubated patients *vs* 9 [IQR 7-11] for those not reintubated; *P* = 0.301). Median peak cardiac troponin was higher for patients who failed extubation (0.05 [IQR 0.02-0.215] vs 0.03 [IQR 0.01, 0.086]). Table 3 summarized therapeutic interventions performed in the overall population and stratified by groups of interest. Reintubated patients had a higher requirement of vasopressor and renal replacement support, but on the day of extubation none were receiving renal replacement and only 2 were requiring small doses (2-5 mcg/min) of norepinephrine which was stopped the following day. These patients also more frequently required salvage therapies such as paralytics and prone ventilation, and more frequently received corticosteroids and therapeutic anticoagulation.

**Table 2.** Baseline Comorbid Conditions and Risk Stratification in Extubated COVID-19 Population.^a^

	All patients (n = 281)	Reintubated (n = 93)	Not reintubated (n = 188)	Significance
Antihypertensive medication	131 (46.6%)	46 (49.5%)	85 (45.2%)	0.501
Diabetes	113 (40.2%)	41 (44.1%)	72 (38.3%)	0.351
CAD	69 (24.6%)	23 (24.7%)	46 (24.5%)	0.961
Heart failure	72 (25.6%)	25 (26.9%)	47 (25.0%)	0.733
Atrial fibrillation	56 (19.9%)	23 (24.7%)	33 (17.5%)	0.156
CVA/TIA	35 (12.5%)	12 (12.9%)	23 (12.2%)	0.873
CKD grade ≥ 3	33 (11.7%)	12 (12.9%)	21 (11.2%)	0.671
Chronic dialysis	14 (5.0%)	5 (5.4%)	9 (4.8%)	0.830
History of VTE	37 (13.2%)	16 (17.2%)	21 (11.2%)	0.159
Chronic lung disease	106 (37.7%)	38 (40.9%)	68 (36.2%)	0.445
History of malignancy	30 (10.7%)	14 (15.1%)	16 (8.5%)	0.094
Peak cardiac troponin	0.03 (0.01-0.105)	0.05 (0.02-0.215)	0.03 (0.01-0.086)	0.011
SOFA score on intubation day	9 (7, 11)	9 (8, 12)	9 (7, 11)	0.301

Abbreviations: CAD, coronary artery disease; CVA, cerebrovascular attack; TIA, transient ischemic attack; CKD, chronic kidney disease; VTE, venous thromboembolism.

^a^ SOFA is reported as median (IQR). Peak troponin is presented as median (IQR); the upper limit of normal for troponin is 0.03 ng/mL. Other numbers represent n (%).

**Table 3.** Interventions in the Overall COVID-19 Population.^a^

	All patients (n = 281)	Reintubated (n = 93)	Not reintubated (n = 188)	Significance
New dialysis	37 (13.2%)	25 (26.9%)	12 (6.4%)	<0.0001
Vasopressors	191 (68.0%)	82 (88.2%)	109 (58.0%)	<0.0001
VC ventilation	159 (56.6%)	49 (52.7%)	110 (58.5%)	0.354
PC ventilation	122 (43.4%)	44 (47.3%)	78 (41.5%)	0.354
Paralytics	97 (34.5%)	43 (46.2%)	54 (28.7%)	0.003
Prone ventilation	93 (33.1%)	40 (43.0%)	53 (28.2%)	0.013
Maximum PEEP	14 (10-16)	14 (12-16)	14 (10-15)	0.225
Corticosteroids	241 (85.8%)	85 (91.4%)	156 (83.0%)	0.057
Therapeutic anticoagulation	171 (60.8%)	72 (77.4%)	99 (52.7%)	<0.0001
Remdesivir	25 (8.9%)	12 (12.9%)	13 (6.9%)	0.097
Tocilizumab	22 (7.8%)	6 (6.5%)	16 (8.5%)	0.545

Abbreviations: VC, volume control; PC, pressure control; PEEP, positive end-expiratory pressure.

^a^ Numbers represent n (%). PEEP is represented as median (IQR).

The number of extubations performed throughout the study period and the proportion of patients requiring reintubation are illustrated in Table 4. Approximately half of all patients (52.3%) were extubated to conventional nasal cannula (NC), whereas little under half (40.6%) required either non-rebreather mask or high flow nasal cannula (NRB or HFNC). A minority (6.8%) were placed on non-invasive positive pressure ventilation (NIPPV), but it is worthwhile to note that early institutional policies discouraged the use of NIPPV due to concern for aerosolization. The incidence of reintubation was significantly smaller among those who only required conventional NC (27 [18.2%]) in the first day following extubation, compared to those who required NRB or HFNC (55 [48.3%]) and NIPPV (11 [57.9%]). The *P*-value for the association was < 0.0001.

**Table 4.** Number of Extubations and Failures Throughout the Study Period.

	Mar	Apr	May	Jun	Jul	Aug	Sep	Oct
All extubated	102	117	19	9	13	11	8	2
Failed extubation	29 (28.4%)	44 (37.6%)	8 (42.1%)	2 (22.2%)	3 (23.1%)	5 (45.5%)	2 (25.0%)	0 (0.0%)

In the overall population, initial intubation occurred on median day 3 (IQR days 1-6); when the 2 groups of interest were compared, patients who failed extubation were initially intubated on median 1 day later (day 4 [IQR days 2-8] *vs* day 3 [IQR days 1-5]; *P* = 0.007). Median days on the ventilator did not differ between the 2 groups (9 days [IQR 5-14 days] in reintubated patients *vs* 8 days [IQR 4.25-15] in those with extubation success; *P* = 0.232).

Extubation failure occurred on median day 3 (IQR days 1.5-6.5) following extubation attempt and 24.7% were reintubated on the same day. Tracheostomy surgery was performed in 24 (25.8%) patients, 29 (31.2%) died inpatient before another extubation attempt or tracheostomy could be performed, and 17 (18.3%) were still alive and intubated at the end of the study period. Less than 1 in 4 patients (23 [24.7%]) eventually underwent successful extubation. Five patients were reintubated 2 or more times in the same hospitalization.

### Reintubation Rate and Relationship to Patient Factors

**Figure 2.**
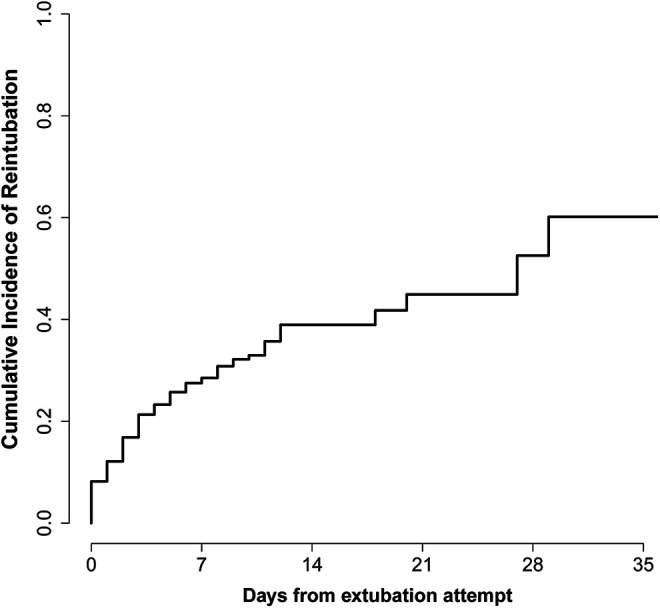
Cumulative incidence function plotting reintubation as event of interest and accounting for death of any cause as competing event.

The cumulative incidence of reintubation as event of interest accounting for death as a competing event is illustrated in Figure 2. The reintubation rate was as high as 8.2% on the day of extubation attempts and rose rapidly over the first 12 days when it plateaued at 39.0%, followed by a slow increase in the subsequent 3 weeks. A multivariate model which included candidate variables suggested by univariate analyses identified the following predictors of extubation failure: advanced age (hazard ratio [HR] 1.04 per 1-year increase, 95% confidence interval [CI] 1.02 -1.06), vasopressor support requirement (HR 1.84, 95% CI 1.04-3.60), renal replacement requirement (HR 2.01, 95% CI 1.22-3.29), the use of paralytics (HR 1.48, 95% CI 1.08-2.25), maximum positive end-expiratory pressure ([PEEP] HR 1.07 per 1-unit increase, 95% CI 1.02 -1.12), and requiring more than conventional nasal cannula in the first 24 hours following extubation (HR 2.19, 95% CI 1.37-3.50). The use of prone ventilation, corticosteroids, therapeutic anticoagulation, remdesivir and tocilizumab were not significantly associated with reintubation risk. Similarly, the number of ventilation days prior to extubation attempt and the month of the pandemic did not show any significant associations in the model.

### Reintubation and Risk of In-Hospital Death

**Figure 3.**
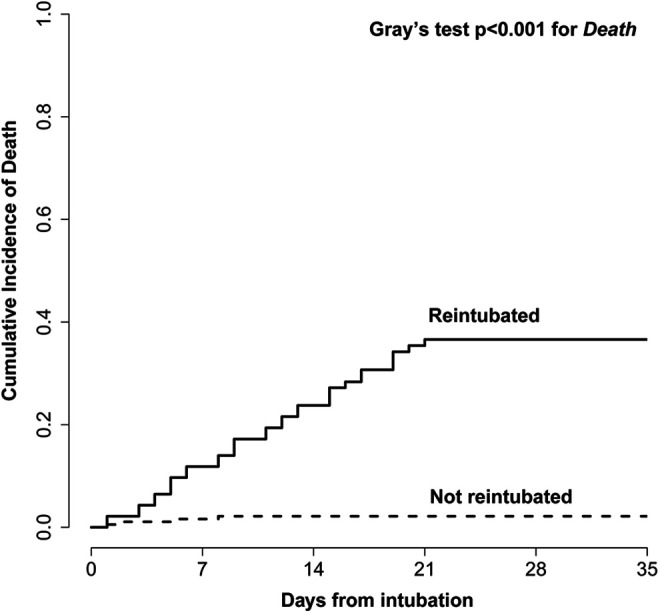
Kaplan-Meier curve plotting survival of extubated COVID-19 patients who were reintubated and those who were not.

A total of 38 patients (13.5%) died during their hospital stay following extubation attempts and most belonged to the extubation failure group (34/93 [36.6%] *vs* 4/188 [2.1%]; *P* < 0.0001). The cumulative risk of death in the reintubation population was significantly higher compared to patients who were successfully extubated (Figure 3). In a multivariate model exploring the risk of in-hospital death as the event of interest, while accounting for discharge alive as competing event, reintubation was strongly associated with a poor outcome (HR 23.2, 95% CI 6.45-83.33). Other predictors of decreased in-hospital survival were a history of ischemic stroke or transient ischemic attack (HR 3.4, 95% CI 1.66-6.98), peak cardiac troponin (HR 1.13 per 1-unit increase, 95% CI 1.08 -1.18), and renal replacement requirement (HR 2.02, 95% CI 1.04-3.92). Vasopressor requirement also correlated with an increased risk of death but did not reach statistical significance (HR 4.50, 95% CI 0.82-24.81). By contrast, paralytic use was associated with improved survival (HR 0.45, 95% CI 0.22-0.90). The use of tocilizumab, remdesivir, therapeutic anticoagulation and corticosteroids did not significantly impact outcome.

## Discussion

In this study, we directly investigate the rate of extubation failure in COVID-19 patients, its determinants and clinical implications. Our main findings are as follows: (1) extubation failure is common in COVID-19 and up to 1 in 3 patients may require reintubation; (2) advanced age, non-pulmonary organ system failure, paralytic use, higher PEEP and higher post-extubation respiratory support requirement predicted extubation failure; (3) experimental therapeutics (corticosteroids, therapeutic anticoagulation, tocilizumab, remdesivir) do not appear to mitigate the risk; and 4) extubation failure was strongly associated with in-hospital mortality.

Previous large studies on non-COVID patients in United States intensive care units have shown a cumulative incidence rate of reintubation of 10% with a median time of 15 hours^6^ and that reintubation is an independent risk factor for mortality in this population.^7^ Whereas most SARS-CoV-2 infections are not severe, approximately 5% of patients develop critical illness, characterized by respiratory failure requiring endotracheal intubation and mechanical ventilation, shock, or multiorgan dysfunction.^8^ Long durations of invasive respiratory support extending beyond 10-14 days have been reported in 50% or more of patients, whereas liberation from the ventilator is attempted in only 48-56%.^1–4^

Compared to other published reports,^1,2,9,10^ extubation was attempted in a smaller number of patients (35%) in our population, but resulted in a higher percentage of reintubations (33%). The median time to the first extubation attempt did not differ between the groups arguing that premature liberation from the ventilator is unlikely to account for failure. Our observation is in line with that of others, who did not find a relationship between the time on endotracheal ventilation prior to the extubation attempts and success or failure.^4^

Half of the patients who required reintubation did so within the first 3 days after extubation attempts and as many as 1 in 4 were reintubated on the first day. The reintubation risk was as high as 8% on the first day and increased rapidly in the first 12 days to approximately 40%. As such, patients appear to be at the highest risk of reintubation during the early phase with the risk plateauing by the end of the second week. Only one other analysis was available for comparison and reported an average time to reintubation of 1.14 days (standard deviation 1.9 days) following extubation attempt, but the small number of events (7/205 [3.4%]) limits interpretability of these findings.^4^

Greater disease severity, frequent need for suctioning post-extubation and ICU-acquired weakness were identified as predictors of extubation failure in non-COVID patients.^11,12^ Clinical markers to predict extubation success or failure are currently unknown in the critically ill COVID-19 population. In our cohort, reintubated patients were significantly older (on average by 8 years) compared to those whose extubation was successful; the comorbidity burden and the mortality risk assessed by the SOFA score on intubation day were similar. In a multivariate model accounting for the risk of in-hospital death, we observed an increase in the risk of reintubation by 4% with every 1-year increase in age, by 84% with the use of vasopressors, and a greater than 2-fold increase with either renal replacement therapy requirement or requirement of more than conventional nasal cannula as respiratory support following extubation. It is likely that the presence of non-pulmonary organ failure represents a marker of continuous or severe systemic inflammation which also continues to affect the respiratory system. The use of paralytics also increased the risk of reintubation by nearly 50%, suggesting that ICU-associated weakness may contribute to extubation failure. Increasing PEEP required to maintain appropriate oxygenation was also associated with reintubation. A higher PEEP may be a cause of barotrauma or may be a sign of more severe alteration in pulmonary biomechanics, both of which could explain a higher rate of reintubation. The use of experimental therapies such as immunosuppression (corticosteroids, tocilizumab), therapeutic anticoagulation or antivirals did not impact the reintubation risk.

Extubation failure was associated with poor outcomes and should be considered a strong marker of disease severity. Among reintubated patients, more than one third died inpatient (37%) and 1 in 4 underwent tracheostomy surgery. Only 1 in 4 were eventually successfully liberated from ventilatory support during their hospital stay. Reintubated patients exhibited a greater than 20-fold increase in the risk of dying inpatient (HR 20.9, 95% CI 6.05-72.3), even after adjusting for multiple patient factors (history of ischemic stroke or transient ischemic attack, need for renal replacement or vasopressors) and after accounting for the competing risk of discharge alive. Interestingly, the use paralytics appeared to decrease the risk of inpatient death. In traditional ARDS, the advantage of using neuromuscular blocking agents remains controversial with the early ACURASYS trial showing a potential mortality benefit,^13^ while the more recent ROSE trial demonstrated no difference with the use of these agents.^14^ A potential explanation for the benefit observed in ACURASYS is that paralytics mitigated ventilator dyssynchrony in the treatment group which had received lighter sedation.^15^ While it may be difficult to extrapolate the findings of prior studies to the ARDS caused by COVID-19, which has been described as atypical in terms of biomechanics and clinical course, ventilator dyssynchrony has been recognized as a frequent problem in the COVID-19 population.^16^ Under these circumstances, neuromuscular blocking agents may increase survival by decreasing ventilator dyssynchrony, but may simultaneously increase the risk of critical illness myopathy and post-extubation respiratory muscle weakness and, thus, increase the rate of reintubation. Reintubation is, therefore, the result of both a higher severity of pulmonary parenchymal disease and of weakened respiratory biomechanics. Our data suggests that, in the event that reintubation does occur, it negates any potential survival advantage associated with paralytic use.

Considering these findings, extubation failure should prompt a re-evaluation of prognosis and treatment goals together with the medical decision maker, as well as potential exploration of a palliative approach.

Future studies should aim to prospectively validate these observations and to evaluate different clinical and biological factors that can better guide the decision of whether or not to attempt extubation in critically ill COVID-19 patients and potentially proceed directly to tracheostomy.

### Limitations

Our analysis has several important limitations as a consequence of its retrospective and observational design. Despite efforts to control for different sources of bias using statistical adjustment, the risk of hidden confounders skewing the results persists. As can be expected with automatically extracted information from electronic medical records, issues of data completeness and correctness can also be the source of bias. The missingness of several variables that merited inclusion was high enough that their impact could not be reliably assessed in multivariate models. Information that would have added nuance to our analysis, including the post-extubation presence of stridor, frequent need for suctioning and ICU-acquired weakness, was not available. However, we included most predictors of adverse outcomes, pulmonary mechanics and therapeutic interventions within our model. We used an established risk stratification scoring system (SOFA) to compare groups at baseline, but this tool has not been validated in COVID-19 patients and its automatic calculation may have resulted in inaccuracies. The timing of the extubation attempt was at the discretion of the multidisciplinary ICU team and our data lacked the granularity to account for heterogeneity in methods of performing spontaneous breathing trials across institutions. As the decision to extubate was not standardized among different hospitals and providers, this wide practice variation could have introduced bias. Another source heterogeneity within the population results from the increase in our clinical experience in the management of COVID-19 throughout the study period. We did attempt to control for this by introducing a time variable representing the month of the pandemic in which extubation was attempted and treatment with antiviral, anti-inflammatory and anticoagulants but all these factors did not impact outcomes after reintubation in our patient group.

Despite its limitations, inherent to the retrospective design, our analysis identifies new areas of study in the treatment of critically ill COVID-19 patients.

## Conclusions

Extubation failure is common in critical illness related to COVID-19 and may affect up to 1 in 3 patients. Predictors of extubation failure are advanced age, higher PEEP and post-extubation respiratory support requirement, the use of paralytics and the presence of non-pulmonary organ failure, such as cardiovascular collapse requiring vasopressor support or renal failure requiring dialysis. When reintubation is necessary it should be considered a strong predictor of mortality and may prompt reconsideration of the goals of care.
